# Milk and dairy products: good or bad for human health? An assessment of the totality of scientific evidence

**DOI:** 10.3402/fnr.v60.32527

**Published:** 2016-11-22

**Authors:** Tanja Kongerslev Thorning, Anne Raben, Tine Tholstrup, Sabita S. Soedamah-Muthu, Ian Givens, Arne Astrup

**Affiliations:** 1 Department of Nutrition, Exercise and Sports, Faculty of Science, University of Copenhagen, Copenhagen, Denmark; 2 Division of Human Nutrition, Wageningen University, Wageningen, The Netherlands; 3 Centre for Food, Nutrition and Health, University of Reading, Reading, UK

**Keywords:** obesity, type 2 diabetes, cardiovascular disease, osteoporosis, cancer, mortality

## Abstract

**Background:**

There is scepticism about health effects of dairy products in the public, which is reflected in an increasing intake of plant-based drinks, for example, from soy, rice, almond, or oat.

**Objective:**

This review aimed to assess the scientific evidence mainly from meta-analyses of observational studies and randomised controlled trials, on dairy intake and risk of obesity, type 2 diabetes, cardiovascular disease, osteoporosis, cancer, and all-cause mortality.

**Results:**

The most recent evidence suggested that intake of milk and dairy products was associated with reduced risk of childhood obesity. In adults, intake of dairy products was shown to improve body composition and facilitate weight loss during energy restriction. In addition, intake of milk and dairy products was associated with a neutral or reduced risk of type 2 diabetes and a reduced risk of cardiovascular disease, particularly stroke. Furthermore, the evidence suggested a beneficial effect of milk and dairy intake on bone mineral density but no association with risk of bone fracture. Among cancers, milk and dairy intake was inversely associated with colorectal cancer, bladder cancer, gastric cancer, and breast cancer, and not associated with risk of pancreatic cancer, ovarian cancer, or lung cancer, while the evidence for prostate cancer risk was inconsistent. Finally, consumption of milk and dairy products was not associated with all-cause mortality. Calcium-fortified plant-based drinks have been included as an alternative to dairy products in the nutrition recommendations in several countries. However, nutritionally, cow's milk and plant-based drinks are completely different foods, and an evidence-based conclusion on the health value of the plant-based drinks requires more studies in humans.

**Conclusion:**

The totality of available scientific evidence supports that intake of milk and dairy products contribute to meet nutrient recommendations, and may protect against the most prevalent chronic diseases, whereas very few adverse effects have been reported.

Several media stories and organisations claim that dairy increases risk of chronic diseases including obesity, type 2 diabetes, cardiovascular disease, osteoporosis, and cancer. Therefore, there is an increasing scepticism among the general consumers about the health consequences of eating dairy products. This is reflected in an increasing consumption of plant-based drinks, for example, based on soy, rice, almond, or oats. Dairy is an essential part of the food culture in the Nordic countries; thus, inclusion of milk and dairy products in the diet may be natural for many Nordic individuals. The major causes of loss of disease-free years in the Nordic countries today are type 2 diabetes, cardiovascular diseases, and cancers. Moreover, the increasing prevalence of obesity greatly increases the risk of these chronic diseases. Given the increasing prevalence of these chronic diseases, it is critically important to understand the health effects of milk and dairy products in the diet. Accordingly, this narrative review presents the latest evidence from meta-analyses and systematic reviews of observational studies and randomised controlled trials on dairy intake (butter excluded) and risk of obesity, type 2 diabetes, cardiovascular disease, osteoporosis, and cancer as well as all-cause mortality.

We aim to answer the key questions: 1) For the general consumer, will a diet with milk and dairy products overall provide better or worse health, and increase or decrease risk of major diseases and all-cause mortality than a diet with no or low content of milk and dairy products? 2) Is it justified to recommend the general lactose-tolerant population to avoid consumption of milk and dairy products? 3) Is there scientific evidence to substantiate that replacing milk with plant-based drinks will improve health?

## Obesity and type 2 diabetes

A large share of the on-going increase in prevalence of type 2 diabetes is driven by the obesity epidemic (1, 2), and it is therefore relevant to assess the role of milk and dairy products for body weight control. Childhood overweight and obesity worldwide is a major contributor to the current obesity epidemic, and childhood obesity frequently tracks into adulthood (3). Therefore, early prevention of childhood obesity is important. A meta-analysis showed that among children in the pre-school and school age, there was no association between dairy intake and adiposity (4). However, there was a modestly protective effect in adolescence. A recent meta-analysis by Lu et al. (5) found that children in the highest dairy intake group were 38% less likely to be overweight or obese compared to those in the lowest dairy intake group. An increase in dairy intake of one serving per day was associated with a 0.65% lower body fat and a 13% lower risk of overweight or obesity.

Milk and dairy products are good sources of high-quality protein. Protein is important during weight loss and subsequent weight maintenance due to the high satiating effect which helps to prevent over-consumption of energy and thereby reduces body fat stores (6, 7). Furthermore, dairy protein is a good source of essential amino acids for muscle protein synthesis and thus helps to maintain the metabolically active muscle mass during weight loss (8). Meta-analyses support that in adults, dairy products facilitate weight loss and improve body composition, that is, reduce body fat mass and preserve lean body mass during energy restriction and in short-term studies (9–11). The effect of an increased dairy consumption on body weight in long-term studies (>1 year) and in energy balance studies is less convincing (10, 11). This is likely due to the opposing effects of dairy on body composition, that is, reduction of fat mass and preservation of lean body mass.

Meta-analyses assessing the role of intake of milk and dairy products on risk of type 2 diabetes have consistently found no or a slight beneficial effect of dairy intake on diabetes risk (12–15). This is consistent with a Mendelian randomisation study using genetic polymorphisms for the lactase gene, which showed that milk intake assessed by lactose tolerance was not associated with risk of type 2 diabetes or obesity (16). The most recent meta-analysis on dairy intake and diabetes incidence included 22 cohort studies with a total of 579,832 subjects and 43,118 type 2 diabetes cases (17). An inverse association between total dairy and yoghurt intake and risk of type 2 diabetes was reported although there was no association with milk intake. The benefits of fermented dairy products (cheese and yoghurt) in relation to type 2 diabetes may be due to their effect on the gut microbiota (18, 19). Other studies have identified that whey protein (primarily in milk and yoghurt) can reduce postprandial plasma glucose concentration in type 2 diabetic subjects (20). This effect may be due to the branched chain amino acids in the whey protein fraction, particularly leucine which has been shown to induce a greater stimulation of glucose-dependent insulinotropic polypeptide (GIP), but not glucagon like peptide 1 (GLP-1), compared to other amino acids (21). The GIP response is possibly a key factor in the higher insulin response and the subsequent lowering of blood glucose seen after whey ingestion, at least in healthy subjects. In addition to the insulinotropic effect of milk, a recent study has indicated that dairy may also improve insulin sensitivity (22).

### Conclusion on obesity and type 2 diabetes

A diet high in milk and dairy products reduces the risk of childhood obesity and improves body composition in adults. This likely contributes to lower the risk of developing type 2 diabetes. Additionally, dairy product consumption during energy restriction facilitates weight loss, whereas the effect of dairy intake during energy balance is less clear. Finally, there is increasing evidence suggesting that especially the fermented dairy products, cheese and yoghurt, are associated with a reduced risk of type 2 diabetes.

## Cardiovascular disease

Low-fat, calcium-rich dairy products are generally considered to lower blood pressure. This was supported by a meta-analysis of six observational studies, whereas no association was found with intake of high-fat dairy products (23). High-fat dairy products are known to increase high density lipoprotein (HDL)- and low density lipoprotein (LDL)-cholesterol concentrations. The latter normally predicts risk of cardiovascular disease (24), but this may depend on the size of the LDL-cholesterol particles. Small, dense LDL particles are more atherogenic than their larger counterparts (25–28) due to their lower affinity for the LDL-receptor and higher susceptibility to oxidation (29). In agreement, some of the fatty acids typically found in milk and dairy products have been associated with less small, dense LDL particles (4:0–10:0 and 14:0 in the diet, and 15:0 and 17:0 in serum phospholipids) (30). In addition, the minerals in milk and dairy products have been shown to attenuate the LDL-response to high-fat dairy intake (31, 32).

Among high-fat dairy products, cheese in particular does not seem to increase LDL-cholesterol to the extent expected, based on the high content of saturated fat (33). When compared to habitual diet with a lower total and saturated fat content (33), or compared to diets with lower total fat content but higher content of high-GI carbohydrates (34, 35), a high intake of cheese was found not to increase LDL-cholesterol. A meta-analysis of randomised controlled trials studying the effect of cheese consumption compared with other foods on blood lipids and lipoproteins showed that cheese caused lower total cholesterol, LDL-cholesterol, and HDL-cholesterol concentrations compared with butter (36). Compared with milk, however, there was no statistically significant difference in blood lipids (32, 37). Several meta-analyses have been conducted on the relationship between intake of milk and dairy products and risk of developing cardiovascular disease. There was no consistent association between milk or dairy intake and cardiovascular disease, coronary heart disease or stroke in a meta-analysis by Soedamah-Muthu et al. (38). In a recent update, including a higher number of prospective cohort studies, there was a significant inverse association between milk intake and stroke, with a 7% lower risk of stroke per 200 ml milk/day, but considerable heterogeneity. Further, stratification for Asian and Western countries showed a more marked reduction in risk in Asian than in Western countries. This is consistent with a previous meta-analysis by Hu et al. (39) showing a non-linear dose–response relationship between milk intake and risk of stroke, with the highest risk reduction of 7–8% with a milk intake of 200–300 ml/day. Also, the meta-analyses by Hu et al. (39) and de Goede et al. (40) both showed an inverse association between cheese intake and stroke, however only borderline significant in the latter. Accordingly, another meta-analysis on dairy and cardiovascular disease found that intake of cheese and milk as well as yoghurt was inversely associated with cardiovascular disease risk (41). A later meta-analysis by Qin et al. (42) found that dairy intake was associated with a 12% lower risk of cardiovascular disease, and 13% lower risk of stroke as compared to individuals with no or a low dairy consumption (42). Likewise, a recent and comprehensive meta-analysis, including 31 cohort studies, suggested that a high dairy intake was associated with a 9% lower risk of stroke, whereas no association was found with total cardiovascular disease or coronary heart disease (43). Moreover, a high intake of cheese was associated with an 8% lower risk of coronary heart disease and a 13% lower risk of stroke. In addition, high plasma levels of the saturated fatty acid C 17:0, which primarily originates from dairy, were found to be associated with a reduced risk of coronary heart disease (44). Finally, a meta-analysis by O'Sullivan et al. (45) found no indication of total dairy intake or any specific dairy product being associated with an increased cardiovascular mortality. Studies are emerging showing that dairy products, particularly the low-fat types, cluster within a healthy dietary pattern (46), and therefore, the risk of residual confounding in the observational studies cannot be ruled out.

In accordance with the latest meta-analyses presented above, the latest Nordic Nutrition Recommendations have concluded that high consumption of low-fat milk products is associated with reduced risk of hypertension and stroke (47).

### Conclusion on cardiovascular disease

The overall evidence indicates that a high intake of milk and dairy products, that is, 200–300 ml/day, does not increase the risk of cardiovascular disease. Specifically, there is an inverse association with risk of hypertension and stroke.

## Bone health and osteoporosis

Milk and dairy products contain a number of nutrients that are required for building strong bones in childhood and for their maintenance during adulthood with the aim to reduce osteoporosis and bone fractures in older age (48). The European Commission has concluded that protein, calcium, phosphorus, magnesium, manganese, zinc, vitamin D, and vitamin K are necessary for maintaining normal bones (European Commission regulation 2012). With the exception of vitamin D, these nutrients are all present in significant amounts in milk and dairy products.

Osteoporosis has been described as a ‘paediatric disease with geriatric consequences’ as low milk, and hence, low mineral intake during childhood and adolescence has been associated with significantly increased risk of osteoporotic fractures in middle and older age, particularly in women (49, 50). A recent study indicated that in children and adolescents, except for those with very low calcium intakes, magnesium intake may be more important than calcium in relation to bone development (51). Calcium intake was found not to be significantly associated with total bone mineral content or density, whereas intake of magnesium and the amount absorbed were key predictors of bone mass. The extent to which these results can be extrapolated to the general population is uncertain, but milk and dairy products are important sources of magnesium and hence important supporters of bone growth during adolescence. In a meta-analysis by Huncharek et al. (52), dairy products, with or without vitamin D supplementation, increased total body and lumbar spine bone mineral content in children with low baseline dairy intake, whereas no effect was found for children with a high baseline dairy intake. Thus, there may be a threshold above which increasing intake of dairy products or dairy-calcium does not additionally benefit bone mineral content or density in children.

In adults, interactions between calcium, phosphorus, protein and vitamin D reduce bone resorption and increase bone formation, thereby attenuating age-related bone loss (53). Possibly due to the complex interaction between nutrients and the multifactorial nature of bone fractures, it has been difficult to establish whether or not a low intake of milk and dairy in adulthood increases the risk of osteoporosis and bone fractures. Hence, to date, meta-analyses have not supported a protective effect of milk and dairy intake in adulthood on risk of osteoporosis and bone fractures (54, 55). Nevertheless a recent systematic review concluded that calcium and dairy are important contributors to bone health in adults (56).

In the 2015–2020 Dietary Guidelines for Americans, it was stated that ‘Healthy eating patterns include fat-free and low-fat (1%) dairy, including milk, yoghurt, cheese, or fortified soy beverages (commonly known as “soymilk”). Those who are unable or choose not to consume dairy products should consume foods that provide the range of nutrients generally obtained from dairy, including protein, calcium, potassium, magnesium, vitamin D, and vitamin A (e.g. fortified soy beverages)’. Although the focus is on achieving the nutrient requirements by foods rather than supplements, plant-based beverages typically contain inorganic chemical forms of calcium, which may actually increase cardiovascular risk (56, 57). As calcium in dairy is organic, milk and dairy products should still be considered the superior sources of calcium (58). Yet, future studies need to address whether or not vitamin D fortification of dairy products is crucial for these to have a positive effect on bone fracture risk.

### Conclusion on bone health and osteoporosis

The present evidence suggests a positive effect of milk and dairy intake on bone health in childhood and adolescence, but with only limited evidence on bone health in adulthood and on the risk of bone fractures in older age.

## Cancer

In population studies, dairy has been associated positively and negatively with various cancers, but most have been based on limited evidence and very few findings remain robust. Dairy products contain a variety of bioactive compounds that could exert both positive and negative effects on carcinogenesis. The positive effects may be related to the content of calcium, lactoferrin, and fermentation products, whereas the negative effects could be linked to the content of insulin-like growth factor I (IGF-1) (59). The World Cancer Research Fund (WCRF) continuously and systematically reviews the evidence on diet and physical activity in relation to prevention of cancer, and specific areas are updated when new evidence has emerged.

Colorectal cancer is the second most common cause of death among cancers in developed countries. Even though colorectal tumourigenesis is a complex process, epidemiological and experimental data indicate that milk and dairy products have a chemopreventive role in the pathogenesis. In the 2011 WCRF report on colorectal cancer, it was concluded that consumption of milk and calcium probably reduces the risk of this cancer (60). Likewise, in meta-analyses, dairy intake has consistently been associated with a decreased risk of colorectal cancer (61, 62) and colon cancer (63). The most recent meta-analysis by Ralston et al. (64) reported 26% lower colon cancer risk in males consuming 525 g of milk per day, whereas no association was found in females.

The link between dairy intake and colorectal cancer is considered to be mainly due to the calcium derived from dairy, with a 24% risk reduction with a dairy-calcium intake of 900 mg/day (65). The proposed mechanisms behind this are calcium binding to secondary bile acids and ionised fatty acids, thereby reducing their proliferative effects in the colorectal epithelium (66). Also, calcium may influence multiple intracellular pathways leading to differentiation in normal cells and apoptosis in transformed cells (67). Accordingly, a number of studies have reported reduced cell proliferation in the colon and rectum with intake of calcium and dairy products (68–72).

In the 2010 WCRF report on breast cancer, it was concluded that the evidence for dairy intake and risk of breast cancer is non-conclusive (73). In accordance with a meta-analysis from 2011 on prospective cohort studies (74), a recent meta-analysis by Zang et al. (75), however, suggested that a high (>600 g/d) and modest (400–600 g/d) dairy intake was associated with a reduced risk of breast cancer (10% and 6%, respectively) compared with a low dairy intake (<400 g/d). Within dairy subgroups, particularly yoghurt and low-fat dairy were found to be inversely associated with the risk of developing breast cancer. As calcium and vitamin D supplementation was previously shown to reduce risk of breast cancer in the Women's Health Initiative (76), these nutrients could be involved in the underlying mechanisms.

According to the 2014 WCRF report on prostate cancer, dairy may be associated with a limited-suggestive increased risk of prostate cancer, but the current evidence is limited (77). However, this conclusion was substantiated by the most recent meta-analysis by Aune et al. (78), which suggested that a high intake of dairy products, milk, low-fat milk, cheese, and calcium were associated with a 3–9% increased risk of prostate cancer. The mechanism behind this was suggested to be an increased circulating concentration of IGF-1, which has been previously shown to be associated with an increased prostate cancer risk (79).

The 2015 WCRF report on bladder cancer suggested that the evidence for milk and dairy on bladder cancer risk was inconsistent and inconclusive (80). Two meta-analyses on milk intake and bladder cancer risk have suggested a decreased risk of bladder cancer with a high intake of milk (61, 81). Others have found no association between milk and dairy intake and risk of bladder cancer risk (82), but none have suggested an adverse effect.

Of the cancer types for which the associations with dairy intake were not presented in the WCRF reports, recent meta-analyses have suggested no association between dairy intake and risk of ovarian cancer (83), lung cancer (84, 85), or pancreatic cancer (86) and an inverse association between dairy intake and risk of gastric cancer in Europe and the United States (87).

### Studies in lactose-intolerant individuals

In a limited number of subjects, potential differences in cancer risk and mortality between lactose-tolerant and lactose-intolerant individuals (self-reported or assessed by polymorphisms for the lactase gene) have been reported under the assumption that lactose-intolerant individuals consume less milk. However, there may also be other differences between these two groups that need to be taken into consideration, for example, genetics, ethnicity, other dietary habits, smoking, physical activity, and socio-economic factors.

Bácsi et al. (88) examined the role of genetically determined differences in the ability to degrade lactose and showed that subjects with deficiencies in the genes coding for lactase (i.e. subjects not drinking milk due to intolerance) had an increased risk of colorectal cancer. This supports the ability of dairy products to reduce colorectal cancer risk and the causality of this relation. In the European EPIC study, the hypothesis that the genetically determined lactose tolerance was associated with elevated dairy product intake and increased prostate cancer risk was examined (89). The study included 630 men with prostate cancer and 873 matched control participants. Dairy product consumption was assessed by diet questionnaires, and intake of milk and total dairy products varied significantly by lactase genotype, with an almost twofold higher intake in lactose-tolerant compared to lactose-intolerant subjects. However, the lactase variant was not found to be significantly associated with prostate cancer risk. This indicates that residual confounding may have biased the associations observed between milk and dairy intake and prostate cancer risk in the observational studies included in a previous meta-analysis (78).

Ji et al. (90) investigated Swedish subjects with self-reported lactose intolerance and found a lower risk of lung, breast, and ovarian cancers compared to lactose-tolerant subjects. Unfortunately, no information about milk intake, or other genetic, ethnic, lifestyle (diet, smoking and physical activity), and behavioural characteristics were reported. Also, self-reported lactose intolerance may not be comparable to genetically determined lactose intolerance. Due to potential bias in the design and the lack of control for known confounders, it is impossible to conclude about the relationship with dairy intake. Also, these findings are in contrast with the additional literature suggesting no or an inverse association between dairy intake and risk of breast cancer (74, 75), ovarian cancer (83, 91), and lung cancer (84, 85).

### Conclusion on cancer

According to WCRF reports and the latest meta-analyses, consumption of milk and dairy products probably protects against colorectal cancer, bladder cancer, gastric cancer, and breast cancer. Dairy intake does not seem to be associated with risk of pancreatic cancer, ovarian cancer, or lung cancer, whereas the evidence for prostate cancer risk is inconsistent. In women, dairy offers significant and robust health benefits in reducing the risk of the common and serious colorectal cancer and, possibly, also the risk of breast cancer. In men, the benefit of the protective effect of milk and dairy on the common and serious colorectal cancer is judged to outweigh a potentially increased risk of prostate cancer.

## All-cause mortality

In medical research, the term ‘all-cause mortality’ implies all causes of death. There are many individual studies reporting that a high consumption of milk and dairy products is associated with decreased mortality (92), unchanged mortality (93), or even increased mortality (94). However, based on meta-analyses of observational cohort studies, there is no evidence to support the view that milk and dairy product intake is associated with all-cause mortality (45, 95). In a meta-analysis, O'Sullivan et al. (45) studied whether intake of milk and dairy products as food sources of saturated fat was related to all-cause mortality, cancer mortality, and cardiovascular mortality. Neither total dairy intake nor intake of any specific dairy products was found to be associated with all-cause mortality. In the most recent meta-analysis including 12 observational studies of milk intake and mortality, there were no consistent associations between milk intake and all-cause or cause-specific mortality (95).

### Conclusion on all-cause mortality

The evidence from observational studies confirms that there is no association between consumption of milk and dairy products and all-cause mortality.

## Comparison of nutrient content and health aspects of milk and plant-based drinks

In recent decades, the market for milk and dairy substitute drinks based on, for example, soy, rice, oats, or almonds has expanded, and calcium-fortified plant-based drinks have become part of the nutrition recommendations as alternatives to milk in several countries, such as the United States, Sweden, Australia, and Brazil. Among the plant-based milk substitutes, soy drink dominates the market in the Western world, but the emerging of other plant-based drinks has influenced the market for soy drink (96).

The nutrient density of plant-based milk substitutes varies considerably between and within types, and their nutritional properties depend on the raw material used, the processing, the fortification with vitamins and minerals, and the addition of other ingredients such as sugar and oil. Soy drink is the only plant-based milk substitute that approximates the protein content of cow's milk, whereas the protein contents of the drinks based on oat, rice, and almonds are extremely low, and the recent review of Mäkinen et al. (96) emphasises the importance of consumer awareness of such low-protein contents. Moreover, there are now cases of severe nutritional deficiencies in children being reported as a result of inappropriate consumption of plant-based drinks (97, 98).

Despite the fact that most of the plant-based drinks are low in saturated fat and cholesterol, some of these products have higher energy contents than whole milk due to a high content of oil and added sugar. Some plant-based drinks have a sugar content equal to that of sugar-sweetened beverages, which have been linked to obesity, reduced insulin sensitivity (99), increased liver, muscle, and visceral fat content as well as increased blood pressure, and increased concentrations of triglyceride and cholesterol in the blood (100, 101). Analyses of several commercially available plant-based drinks carried out at the Technical University of Denmark showed a generally higher energy content and lower contents of iodine, potassium, phosphorus, and selenium in the plant-based drinks compared to semi-skimmed milk (102). Also, rice drinks are known to have a high content of inorganic arsenic, and soy drinks are known to contain isoflavones with oestrogen-like effects. Consequently, The Danish Veterinary and Food Administration concluded that the plant-based drinks cannot be recommended as full worthy alternatives to cow's milk (102), which is consistent with the conclusions drawn by the Swedish National Food Agency (103).

The importance of studying whole foods instead of single nutrients is becoming clear as potential nutrient–nutrient interactions may affect the metabolic response to the whole food compared to its isolated nutrients. As the plant-based drinks have undergone processing and fortification, any health effects of natural soy, rice, oats, and almonds cannot be directly transferred to the drinks, but need to be studied directly. Only a few studies have compared the effects of cow's milk with plant-based drinks as whole foods on disease risk markers (104–108). However, none of these have included commercially available drinks or disease endpoints. Therefore, the evidence is currently insufficient to conclude that plant-based drinks possess health benefits above those of milk and dairy products. Until more research has been conducted and a scientifically sound conclusion can be drawn, health authorities should be cautious about recommending plant-based drinks as acceptable substitutes to cow's milk for the general population.

### Conclusion on nutrient content and health aspects of milk and plant-based drinks

Cow's milk and plant-based drinks are completely different products, both regarding nutrient content and presumably also health effects. Although there are concerns about children consuming the low-protein drinks, further evidence-based assessment of the nutritional and health value of the plant-based drinks must await more studies in humans.

## Answers to the key questions

Key question 1: For the general consumer, will a diet with milk and dairy products overall provide better or worse health, and increase or decrease risk of major diseases and all-cause mortality than a diet with no or low content of milk and dairy products?

**Fig. 1:**
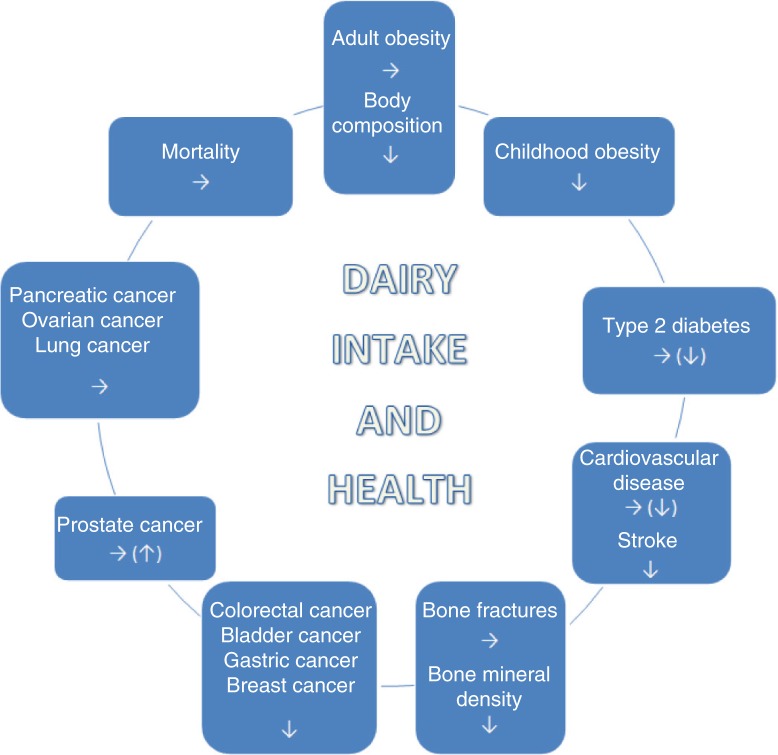
Overall effect/association between dairy product intake and health outcomes. ↓ favourable effect/association; ↑adverse effect/association; → no effect/association.

Consumption of dairy products is associated with an overall reduced risk of cardiometabolic diseases and some cancers, whereas only very few adverse effects have been reported (Fig. 1). Dairy products may therefore have the potential to reduce the burden of the most prevalent chronic diseases in the population and to substantially reduce the health care costs for society (109). Consumption of dairy products is part of the dietary recommendations in several nations, for example, Sweden, Denmark, and United States. A general recommendation to reduce the intake of dairy products in individuals who actually tolerate them may be counterproductive for health and could therefore increase health care expenses. However, more emphasis should be on the foods which dairy replaces in the diet. In addition, as most of the conducted meta-analyses are on observational data, residual confounding cannot be ruled out, and it is also possible that milk and dairy intake in these studies could be just a marker of diets of higher nutritional quality.

Key question 2: Is it justified to recommend the general lactose-tolerant population to avoid the consumption of milk and dairy products?

In the Nordic countries, as few as 2% of the population has primary lactase deficiency and can be classified as lactose-intolerant individuals (110). Yet, most lactose-intolerant adults can tolerate one glass of milk or a scoop of ice cream. Cheeses have negligible lactose contents, and the lactose in yoghurt is digested more efficiently than other dairy sources due to the bacterial lactase present in yoghurt which facilitates lactose digestion (111). Therefore, fermented dairy products, that is, yoghurt and most cheeses (cottage cheese, as well as soft and hard cheeses), can be tolerated by lactose-intolerant individuals without symptoms (111, 112).

The same applies to cow's milk protein allergy that typically occurs in 0.1–2.0% of children in the Nordic countries and Europe (113). Among children with verified cow's milk-specific IgE who were re-evaluated 1 year after diagnosis, 69% tolerated cow's milk at re-evaluation (114). Thus, the condition is generally seen to resolve in children. To warn the general population against dairy consumption based on rare milk allergies would be equivalent to warn against foods, such as peanuts or seafood due to the fact that a small subset of the population is allergic to these foods.

Key question 3: Is there scientific evidence to substantiate that replacing milk and dairy products with plant-based drinks will improve health?

Cow's milk and plant-based drinks are not nutritionally comparable foods. As only a few studies have investigated the health effects of replacing cow's milk with plant-based drinks and none have focused on commercially available drinks or on disease endpoints, the effect of this replacement can only be speculated on. There have, however, been individual cases reporting illness in children consuming low-protein plant-based drinks, but an evidence-based final assessment of the health value of plant-based drinks compared to cow's milk must await more studies in humans.

## Overall conclusions regarding intake of milk and dairy products and health

Our review of the totality of available scientific evidence supports that intake of milk and dairy products contributes to meeting nutrient recommendations and may protect against the most prevalent, chronic non-communicable diseases, whereas very few adverse effects have been reported.
